# The soleus muscle flap for coverage of mid-third leg defects: a Jamaican experience

**DOI:** 10.1093/jscr/rjab369

**Published:** 2021-11-11

**Authors:** Geoffrey Williams, Gabriella Diaz

**Affiliations:** Division of Plastic and Reconstructive Surgery, Department of Surgery, Cornwall Regional Hospital, Montego Bay, Jamaica; Division of Plastic and Reconstructive Surgery, Department of Surgery, Cornwall Regional Hospital, Montego Bay, Jamaica

## Abstract

Motor vehicle accidents are a major public health problem in Jamaica, representing the leading cause of death in adolescents and young adults and consuming a large part of the health care budget, as surviving victims usually require surgical intervention and prolonged hospital stays.

Patients with mid-third compound tibial fractures present a particularly difficult surgical challenge due to the difficulty in achieving soft-tissue coverage of the bone in this area, hence the need for extended hospitalization in order to prevent infection.

We have used a Hemi-Soleus Muscle Flap to achieve coverage of these difficult wounds, thereby eliminating the risk of osteomyelitis, reducing hospital stays and returning patients to productive lives in a much shorter period.

## INTRODUCTION

In Jamaica, road traffic victims account for almost half of hospital bed occupancy on surgical wards and place a severe burden on the health care budget of the country [1, 2].

In the western part of the island with its flat terrain, the use of motorized cycles is widespread and this contributes greatly in making these vehicles responsible for over 30% of road fatalities on the island. Not surprisingly, the vast majority of accident victims are males in the 20–29 age cohort [1, 3, 4].

The tibial shaft is narrowest at the junction of its middle and inferior thirds, which is the most frequent site of fracture. Unfortunately, this area of the bone also has the poorest blood supply and the anteromedial portion of the tibia is covered by skin and subcutaneous fat only. These factors lead to many instances of bone exposure and delayed healing which require specialized soft-tissue coverage [5, 6, 7]. A local muscle flap such as the hemisoleus is ideal, especially in jurisdictions like ours where access to microsurgical reconstruction is limited. The patients of this case series were managed by the Plastic and Reconstructive Surgery Service at a hospital.

## CASE SERIES

### Demographics

This descriptive study from 2016 to 2019 comprises 27 patients on whom the hemisoleus muscle flap was performed for mid-third leg defects. Except for one male who was 41, all the others were in the age range of 20–33 years. A lone female was aged 8 years.

### Medical history

All patients had bone exposure with a type 3B fracture. A total of 25 of the 27 patients (93%) sustained their injuries in motorcycle accidents. Two male patients suffered gunshot wound and the lone female was struck on the leg; these cases will be presented in detail.

### Surgical technique and management overview

An incision is made on the medial aspect of the leg commencing ~10 cm below the popliteal fossa extending to the achilles tendon. The skin marking for this is the deep border of the gastrocnemius muscle. The soleus and gastrocnemius muscles are identified and separated. The hemisoleus is then divided from its insertion at the achilles tendon and dissected cephalad to allow for a sufficient arc of rotation to cover the defect. Typically two sets of perforating vessels are divided to achieve this. The hemisoleus is then inset into the defect and sutured in place using absorbable sutures. Except for the lone female who received a full-thickness skin graft (FTSG), all patients received split-thickness skin grafts (STSG) to cover the muscle. Tie-over silk sutures are employed to bolster the dressing and protect the graft.

The donor incision is closed in layers and a penrose drain placed in the depth of the wound. All patients received 5 to 7 days of intravenous antibiotics perioperatively and post-operatively. The average time to discharge post-surgery was 10 days and patients were then seen at least weekly for the first 2 months in the out-patient clinic and then monthly to quarterly up to a year.

## CASES FROM THE COHORT

### Case 1

**Figure 1:**
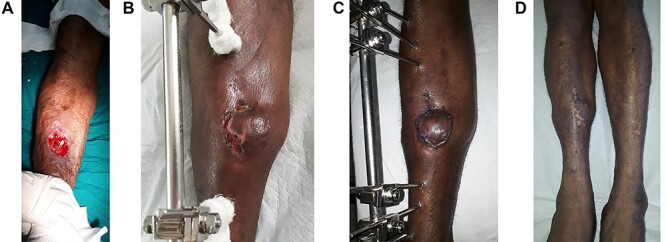
**A:** The wound defect prior to the raising of the hemisoleus muscle flap and STSG. **B:** At 1 month post-operation with minimal skin graft loss laterally. **C:** At 2 months post-operation; completely healed. **D:** At 2 years post-operation; atrophy of the hemisoleus muscle is appreciated.

The first case is that of a 30 year-old man with no chronic illnesses who presented with a gunshot wound to his right leg, fracturing his mid-tibia. Of significance in the history, he had sustained fractures of the right tibia and fibula 6 years previously. On examination of his right anterolateral leg, there was a 7 cm × 5 cm wound and a comminuted fracture of the tibia was apparent. Initial treatment included antibiotics, analgesics, wound irrigation and the placement of a Plaster of Paris Back Slab and the wound dressed daily with an antibiotic ointment. In a joint operation with the Orthopaedic team, an external fixator was placed in the tibia and a soleus muscle flap raised to cover the exposed bone 19 days post-injury. His post-operative course was uneventful except for some early minor skin graft loss which went on to complete healing (Fig. 1).

### Case 2

**Figure 2:**
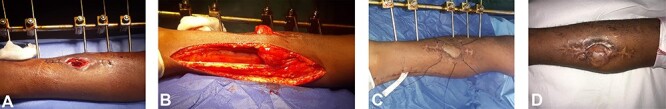
**A:** The wound defect prior to the raising of the hemisoleus muscle flap. **B:** The medial incision, demonstrating that the hemisoleus muscle has been divided at its insertion, raised and will cover the defect, but it is not yet inset. **C:** The hemisoleus muscle has been inset, sutured in place and covered with an FTSG. Note the drain *in situ* within the now closed medial incision. Silk ties were employed to bolster and protect the graft. **D:** Completely healed now with an STSG at 1 week shy of 6 months.

This case is that of the only female in the study; an 8 year-old with Sickle Cell Disease (HBSC). She sustained a type 3 B fracture with subsequent development of osteomyelitis of the right tibia as a result of being hit by a bicycle. She was initially managed by the Orthopaedic team at another institution prior to the referral to Plastic Surgery. Three months post-injury there was a joint surgery between Orthopaedic and Plastic Surgery teams. Debridement of the bone was first performed followed by elevation of a soleus muscle flap which was covered with an FTSG. Approximately 3 weeks post-operation, it was reported that she had developed a flaring osteomyelitis of the right tibia and returned to surgery for bony debridement and that the flap had been lost. However, upon later examination under general anaesthesia by the Plastic Surgery team, the presence of a healthy soleus muscle was noted and this was readvanced to cover the tibia. The muscle was then covered with an STSG and she went on to complete healing (Fig. 2).

## DISCUSSION

The soleus and gastrocnemius muscles make up the three-headed triceps surae. The triceps surae in addition to the plantaris muscle comprise the superficial muscles of the posterior compartment of the leg which is innervated by the tibial nerve. The soleus muscle which resembles a sole is located deep to the gastrocnemius muscle. The soleus muscle flap is classified as a type II according to Mathes and Nahai Classification. It is supplied by perforators of the popliteal, posterior tibial and peroneal arteries. The constant feature of the medial part of the soleus muscle being supplied throughout its whole length by perforators arising from the posterior tibial artery makes the medial part of the muscle reliable, as a proximally or distally based flap. There is an avascular plane in the distal part of the muscle along its deep surface between the perforators of the posterior tibial and peroneal arteries. The average distances of the lower perforators arising from the posterior tibial artery are 6.5, 11.6 and 16.8 cm from the medial malleolus [8]. The lateral approach requires the dissection of an additional peroneal compartment; the risk of damaging the common peroneal nerve is always present [9]. Since the soleus is a bipennate muscle with independent blood supply of each half, using the hemisoleus retains its function, increases its arc of rotation and makes it easy to orientate for coverage of any defect or shape. Due to this geometrical advantage, it is a superior option than the entire soleus [10]. Pertaining to the patient highlighted in Fig. 1, the hemisoleus muscle flap was performed 19 days post-injury. As noted in other studies the dissection and separation of his soleus muscle proved challenging due to chronic inflammation with dense fibrosis [9]. However, the hemisoleus flap was still successfully raised and sutured into the defect.

The second case involved osteomyelitis and the soleus flap was performed 3 months post the initial insult. This case posed more of a challenge due to the longer duration of a chronic wound, leading to difficulty in the recognition and separation of the muscles. Although the question may be posed as to whether to contemplate a muscle flap in the presence of an infected wound or to cover osteomyelitis, it has been shown that it can be safely performed. The critical point to determine is whether and how much drainage is flowing from the bone. Additionally, it can be utilized on a contaminated, however not a grossly infected, wound; debridement is warranted before flap transfer. The hemisoleus muscle flap is an ideal option to cover wounds in the middle third of the leg due to trauma, ulcers and osteomyelitis for a number of reasons. The soleus muscle has a robust vasculature especially from the posterior tibial artery providing excellent blood support and circulation to the tissues. It aids in controlling infection because the flap allows for the supply of antibiotic substances. The soleus muscle also provides a good environment for osteogenesis, due to the expression of transforming growth factor-B, interleukine-6 and fibroblast growth factor-2 [9, 11, 12].

In conclusion, the resilience of the soleus muscle flap has been demonstrated and this is in keeping with a number of documented studies since 1973 [9, 11, 12]. Early reconstruction by soft tissue to cover exposed bone significantly reduces the risk of infection, nonunion and subsequent amputation. Due to their vascularity, muscle flaps tolerate and even help combat infection. Therefore, chronic infection in the recipient area should not be considered a contraindication to the transposition of a muscle flap, but rather should be seen as a means of aiding to combat infection.

## References

[1] Tazhmoy C , McGrowder D. Road traffic injury epidemic in Jamaica: implications for governance and public policy. Asian Soc Sci 2008;4:182–91.

[2] Crandon IW , Harding HE, Cawich SO, McDonald AH, Fearron-Boothe D. Motorcycle accident injury profiles in Jamaica: an audit from the University Hospital of the West Indies. Int J Inj Contr Saf Promot 2009;16:175–8. 19941216 10.1080/17457300903024236

[3] Hall A . Rebellious motorcyclists paying with their lives. Jamaica Observer 2019. https://www.jamaicaobserver.com/news/rebellious-motorcyclists-paying-with-their-lives-more-than-50-dead-in-road-mishaps-so-far-this-year_165753 (27 May 2019, date last accessed).

[4] Reports and statistics . Road safety unit. https://www.mtw.gov.jm/roadsafety/index.php/en/safety/reports-and-statistics (2 July 2021, date last accessed).

[5] Sinnatamby CS . Last’s anatomy regional and applied, Vol. 145-6. Philadelphia: Elsevier, 2006, 176.

[6] Moore KL , Dalley AR, Agur AM. Clinically oriented anatomy, Vol. 520-2. Baltimore: Lippincott Williams & Wilkins, 2010, 527.

[7] Thorne CH , Chung KC, Gosain AK, Gurtner GC, Mehrara BJ, Rubin JP, et al. Grabb and smith’s plastic surgery, Vol. 942. Philadelphia: Lippincott Williams & Wilkins, 2014.

[8] El Zawaawy EMM , El Sekily M. An anatomical study of the blood supply of the soleus. Alexandria Journal of Medicine 2012;48:315–21.

[9] Ahmad I , Akhtar S, Rashidi E, Khurram MF. Hemisoleus muscle flap in the reconstruction of exposed bones in the lower limb. J Wound Care 2013;22:635–42. 24225604 10.12968/jowc.2013.22.11.635

[10] Ata-ul-Haq TMN , Malik FS, Khalid K, Riaz A, Mehrose MY, et al. Hemisoleus muscle flap, a better option for coverage of open fractures involving middle third of tibia. J Ayub Med Coll Abbottabad 2009;21:154–8. 21067051

[11] Satter T , Khan MN, Awwal R. Soleus muscle flap for the coverage of pre-tibial defect of middle third of leg. Bangladesh Journal of Plastic Surgery 2013;4:10–5.

[12] Pers M , Medgyesi S. Pedicle muscle flaps and their applications in surgery of repair. Br J Plast Surg 1973;26:313–21. 4759972 10.1016/s0007-1226(73)90032-5

